# Superior Microwave Absorption Properties Derived from the Unique 3D Porous Heterogeneous Structure of a CoS@Fe_3_O_4_@rGO Aerogel

**DOI:** 10.3390/ma13204527

**Published:** 2020-10-13

**Authors:** Hui Liu, Ling Li, Xinxin Wang, Guangzhen Cui, Xuliang Lv

**Affiliations:** 1 Graduate School, The Army Engineering University of PLA, Nanjing 210007, China; liuhh1005@163.com (H.L.); 18260093995@163.com (X.W.); 2 Engineering College of Field Engineering, The Army Engineering University of PLA, Nanjing 210007, China; xllu1957@126.com

**Keywords:** microwave absorption, rGO aerogel, CoS, porous structure

## Abstract

A novel CoS@Fe_3_O_4_@rGO aerogel with a unique 3D porous heterostructure was prepared via the solvothermal method, in which cobalt sulfide (CoS) microspheres embedded with Fe_3_O_4_ nanoparticles were randomly scattered on reduced graphene oxide (rGO) flakes. The introduction of magnetic Fe_3_O_4_ nanoparticles and rGO regulated the impedance matching, and the excellent electromagnetic wave (EMW) absorption capability of the CoS@Fe_3_O_4_@rGO aerogel could be attributed to optimal dielectric loss and abundant conductive networks. The results demonstrated that the minimum reflection loss (RL) value of CoS@Fe_3_O_4_@rGO aerogel was −60.65 dB at a 2.5 mm coating thickness with an ultra-wide bandwidth of 6.36 GHz (10.24–16.6 GHz), as the filler loading was only 6 wt%. Such a lightweight CoS@Fe_3_O_4_@rGO aerogel with an outstanding absorbing intensity and an ultra-wide effective absorption bandwidth could become a potential EMW absorber.

## 1. Introduction

In recent years, wireless communication technology and radar detection have been widely used in daily life and military fields [1,2]. With a lot of attention being paid to the increasing pollution of serious electromagnetic waves (EMWs), high-performance absorbing materials have come into people’s field of vision, which have the characteristics of lightweight, strong absorption, broad absorption bandwidth, thin matching thickness, and so on [3,4,5]. The attenuation capability of EMWs can be characterized by their electromagnetic parameters; it is critical to design materials with good microstructure and phase composition [6,7]. According to reports in recent years, common wave-absorbing materials include ferrites [8], carbon nanomaterials [9], conductive polymers [10], and transition metal oxides [11]. Because of its low price, good stability, and high permeability, Fe_3_O_4_ stands out from many other EMW-absorbing materials and has been widely studied [12,13,14,15,16,17]. With its unique nanostructure and good magnetic loss properties, Fe_3_O_4_ shows great EMW absorption potential; however, defects such as easy corrosion, high density, and narrow absorption frequency band seriously limit its practical application. 

As the thinnest two-dimensional materials at present, graphene oxide (GO) and reduced graphene oxide (rGO) exhibit superior properties of lightweight, a large and specific surface area, good chemical stability, and mechanical performance [18,19,20]. At present, rGO has excellent performance in energy storage [21], catalysis [22], stealth technology [23], and other fields [24,25,26]. Owing to its high electron mobility and remarkable conductivity, electrons migrate on the surface of rGO to form a conductive network, which gives full play to the role of dielectric loss [27]. In addition, the abundant defects and functional groups in rGO may cause polarization relaxation and dipole relaxation, which are conducive to increasing the absorption of EMWs [28]. However, due to poor impedance matching, the absorbing performance of pure rGO is not ideal. Therefore, decorated with Fe_3_O_4_ magnetic nanoparticles, the impedance matching and EMW absorption capacity of rGO-based composites could be significantly improved. For instance, Li et al. reported nitrogen-doped GO nanosheets assembled by Fe_3_O_4_ nanoparticles, and the optimal reflection loss (RL) could achieve −65.3 dB at 6.8 GHz and 3.4 mm [29]. Zhu et al. prepared Fe_3_O_4_/rGO composites, and the optimal RL was −45 dB at 8.96 GHz, the absorber thickness was 3.5 mm, while the corresponding effective absorbing frequency bandwidth was 3.2 GHz [30]. However, it is worth noting that Fe_3_O_4_ nanoparticles tend to agglomerate on the surface of rGO due to magnetic attraction, affecting the absorption of incident EMWs. It was found that the introduction of sulfide into the rGO–Fe_3_O_4_ composites could significantly reduce the agglomeration of ferrite nanoparticles and could enhance the EMW absorption performance [31]. In our previous studies, cobalt sulfide (CoS) exhibited impressive wave-absorbing properties. Hence, it is feasible to synthesize a CoS@Fe_3_O_4_@rGO composite, which can not only reduce the density of magnetic materials, but also has excellent impedance matching and a strong wave-absorbing ability.

Herein, we prepared a novel CoS@Fe_3_O_4_@rGO aerogel via the solvothermal method. The microstructure, elemental composition, EMW absorption performance, and possible mechanism of the products were carefully investigated. The morphology of the CoS@Fe_3_O_4_@rGO aerogel indicated that CoS microspheres embedded with many Fe_3_O_4_ nanoparticles were randomly scattered on the rGO flakes, forming a unique 3D porous heterostructure. Moreover, due to the introduction of magnetic Fe_3_O_4_ nanoparticles, the impedance matching of the material could be effectively optimized. The outstanding EMW-absorbing performance of the CoS@Fe_3_O_4_@rGO aerogel was revealed.

## 2. Materials and Methods 

### 2.1. Material Preparation

The graphite powder (200 mesh) was received from XFNANO (XFNANO Materials Tech Co., Ltd., Nanjing, China). Cobalt chloride hexahydrate (CoCl_2_·6H_2_O) and sodium acetate (NaAc) were purchased from Aladdin Technology Co. Ltd. (Shanghai, China). Thioacetamide (TAA), absolute ethanol (CH_3_CH_2_OH), ethylene glycol (EG), trisodium citrate (Na_3_C_6_H_5_O_7_·2H_2_O), ascorbic acid, and ferric chloride hexahydrate (FeCl_3_·6H_2_O) were provided by Sinopharm Chemical Reagent Factory (Shanghai, China). All of the above materials were of analytical grade and used directly.

### 2.2. Fabrication of the Fe_3_O_4_ Nanoparticles

First, 4.3 g of FeCl_3_·6H_2_O and 1 g of Na_3_C_6_H_5_O_7_·2H_2_O were dispersed in 70 mL of EG. Then, the mixed solution was stirred continuously for 30 min. Four grams of NaAc was added into the suspensions, followed by sonication for 30 min until a uniform solution was formed. Afterward, the solution was sealed and maintained at 200 °C for 10 h in a 200 mL Teflon-lined autoclave. After cooling to room temperature, the black precipitates were collected by centrifuge and washed alternately with deionized water and absolute ethanol, and then dried at 50 °C for 12 h.

### 2.3. Preparation of the CoS@Fe_3_O_4_ Microspheres

CoCl_2_·6H_2_O (2.5 mmol) and as-prepared Fe_3_O_4_ (0.58 g) were dissolved in 25 mL of absolute ethanol, respectively, and the two solutions were mixed together at room temperature and then given an ultrasound treatment for 30 min. TAA (5 mmol) was dissolved in 50 mL of absolute ethanol and added dropwise into the above suspension, followed by ultrasonic treatment for 30 min. Then the obtained solution was transferred into a reaction vessel, sealed, and kept at 160 °C for 24 h. The final products were washed with ethanol and deionized water and dried in a vacuum oven at 50 °C for 10 h.

### 2.4. Fabrication of the CoS@Fe_3_O_4_@rGO Aerogel

The graphene oxide (GO) was obtained by a modified Hummers method [32,33]. GO (40 mg) was dispersed in 70 mL of deionized water and then placed in an ultrasonic bath to form a homogeneous suspension. The as-prepared CoS@Fe_3_O_4_ microspheres were added into the above suspension and mechanically stirred for 30 min. Afterward, ascorbic acid (72 mg) was dissolved in the resulting mixture, followed by ultrasonic treatment for 5 min. The final product was moved and sealed into a Teflon-lined autoclave (100 mL), and then maintained at 120 °C for 4 h. Finally, the CoS@Fe_3_O_4_@rGO aerogel was obtained after freeze-drying for 24 h. 

### 2.5. Material Measurement 

The microstructure information of the products was revealed by a scanning electron microscope (SEM; Quanta 250, FEI, Hillsboro, OR, USA) and a transmission electron microscope (TEM; JEM-2100F, JEOL, Tokyo, Japan). The elemental states were measured by energy-dispersive X-ray spectroscopy mapping (EDS; XFlash 5030T, BRUKER, Leipzig, Germany). The crystal structures were investigated by a D-MAX-2500 X-ray powder diffractometer (XRD, Rigaku, Beijing, China). Raman spectroscopy was characterized by a confocal Raman spectrometer (RM 2000, Renishaw PLC, London, UK). The surface properties of the materials were tested using X-ray photoelectron spectroscopy (XPS; ESCALAB 250XI, Shanghai, China). At room temperature, the products were blended with paraffin wax at different loading ratios (i.e., 4, 6, and 8 wt%), and then pressed into a coaxial ring ($\Phi_{in}\;$= 3.04 mm, $\Phi_{out}\;$= 7.0 mm). For convenience, the corresponding products were coded as S1, S2, and S3, respectively, and the electromagnetic parameters of the products were calculated using a vector network analyzer (VNA, Agilent E8363B, Palo Alto, CA, USA) ranging from 2 to 18 GHz.

## 3. Results

### 3.1. Characterization of the Products 

**Figure 1:**
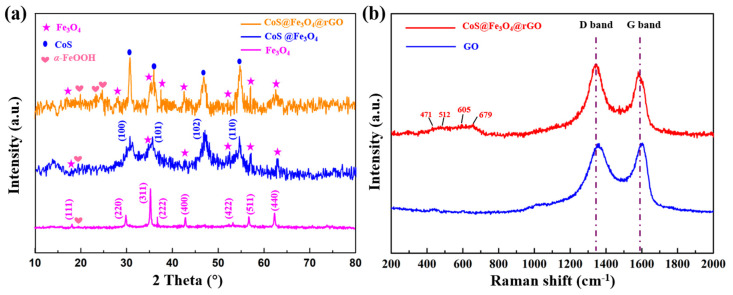
(**a**) X-ray powder diffraction (XRD) curves of Fe_3_O_4_, CoS@Fe_3_O_4_, and CoS@Fe_3_O_4_@rGO; (**b**) Raman spectra of graphene oxide (GO) and CoS@Fe_3_O_4_@rGO.

The X-ray diffraction analysis of Fe_3_O_4_, CoS@Fe_3_O_4_, and CoS@Fe_3_O_4_@rGO is shown in Figure 1a. The characteristic diffraction peaks located at 2θ = 18.34°, 30.09°, 35.35°, 37.03°, 43.08°, 53.30°, 56.77°, and 62.43° are consistent with the (111), (220), (311), (222), (400), (422), (511), and (440) planes, respectively, which reveals the formation of the spinel Fe_3_O_4_ (JCPDS no. 19-0629) [34]. In addition, the peaks located at 2θ = 20.12°, 24.39°, and 25.44° could correspond to the (110), (120), and (310) of *α*-FeOOH (JCPDS card no. 9003076). This is because of uneven heating in the hydrothermal process, resulting in part of the residual FeOOH [35,36]. The peaks assigned to (100), (101), (102), and (110) indicate hexagonal CoS (JCPDS no. 42-0826) [37]. No diffraction peaks of rGO were detected because the reaction process destroyed the structure of rGO, resulting in its low crystallinity. Raman spectroscopy was used to observe the degree of crystallinity and graphitization of the materials. As can be seen in Figure 1b, the peaks located at 1361.57 cm^−1^ and 1592.95 cm^−1^ correspond to the D and G bands of GO, respectively. As regards CoS@Fe_3_O_4_@rGO, the D and G bands are located at 1342.5 cm^−1^ and 1579.07 cm^−1^. The D peak is related to the lattice defects of graphene sheets, while the G peak is induced by the vibration of hybrid carbon atom sp^2^ [38]. The degree of carbon crystallization is usually characterized by the relative strength value of D and G peaks (I_D_/I_G_), and the crystallization is inversely proportional to the I_D_/I_G_ value [39]. The results show that the I_D_/I_G_ value of CoS@Fe_3_O_4_@rGO increased from 0.93 to 1.08, indicating more defects within the composite. By contrast, it was demonstrated that most of the oxygen in functional groups had been removed, the disorder of carbon in CoS@Fe_3_O_4_@rGO increased, and the GO was successfully reduced to rGO during the hydrothermal process [40]. Additionally, the four characteristic peaks between 400 and 800 cm^−1^ may be attributed to CoS, and the low crystallinity of CoS is probably due to the low-temperature preparation [41,42].

**Figure 2:**
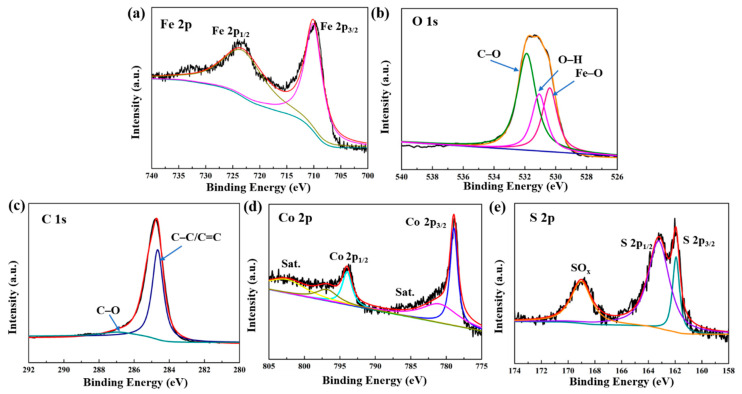
X-ray photoelectron spectroscopy (XPS) spectra of CoS@Fe_3_O_4_@rGO: (**a**) Fe 2p, (**b**) O 1s, (**c**) C 1s, (**d**) Co 2p, and (**e**) S 2p.

To further observe the detailed surface electronic state of CoS@Fe_3_O_4_@rGO, the XPS analysis is given in Figure 2. The Fe 2p spectrum curve can be fitted by two peaks located at 710.6 eV and 724.2 eV, which are in agreement with Fe 2p_3/2_ and Fe 2p_1/2_ (Figure 2a) [43]. A shakeup satellite peak located at approximately 719 eV cannot be observed, which means that the iron oxide in the composite is Fe_3_O_4_, not γ–Fe_2_O_3_ [29]. In Figure 2b, the O 1s spectrum displays a stronger peak of C–O at 532.3 eV compared to the O–H peak at 531.2 eV and the Fe–O peak at 529.9 eV. These oxygen vacancy defects may be beneficial to the absorption of EMWs [44,45]. As for C 1s (Figure 2c), the peaks at 284.67 eV and 286.2 eV are related to the C–C/C=C and C–O bonds. Figure 2d represents the spectra of Co 2p, in which the peaks indexed to Co 2p_3/2_ and Co 2p_1/2_ are at 779.8 eV and 794.5 eV, respectively. Moreover, the peaks at 785.3 eV and 805.2 eV in the Co 2p spectrum are ascribed to the satellite binding energies, which can be attributed to the oxidation of metal cobalt in the air [46]. With respect to the S 2p spectra (Figure 2e), two peaks corresponding to S 2p_3/2_ and S 2p_1/2_ are located at 161.92 eV and 163.22 eV [47], respectively, and the other strong binding energy at 168.7 eV may be due to SO_x_, which can be caused by partial sulfur oxidation [48]. Hence, the XPS results further confirm the formation of a CoS@Fe_3_O_4_@rGO composite.

**Figure 3:**
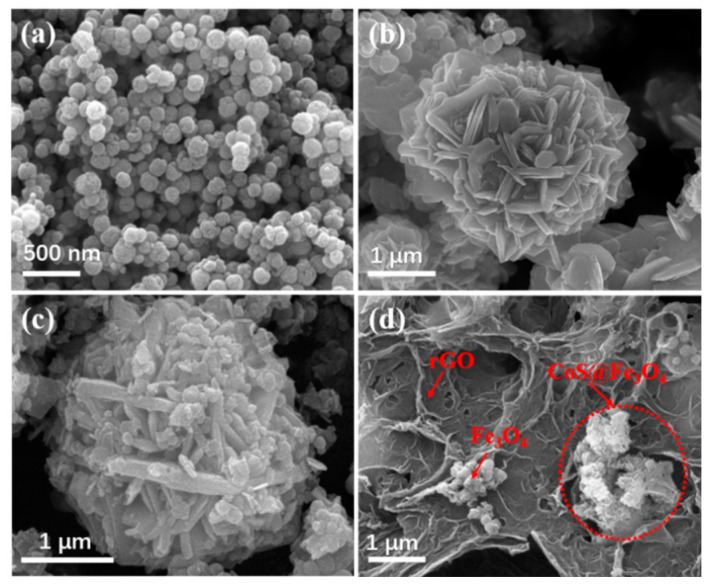
Scanning electron microscopy (SEM) images of (**a**) Fe_3_O_4_, (**b**) CoS, (**c**) CoS@Fe_3_O_4_, and (**d**) CoS@Fe_3_O_4_@rGO.

**Figure 4:**
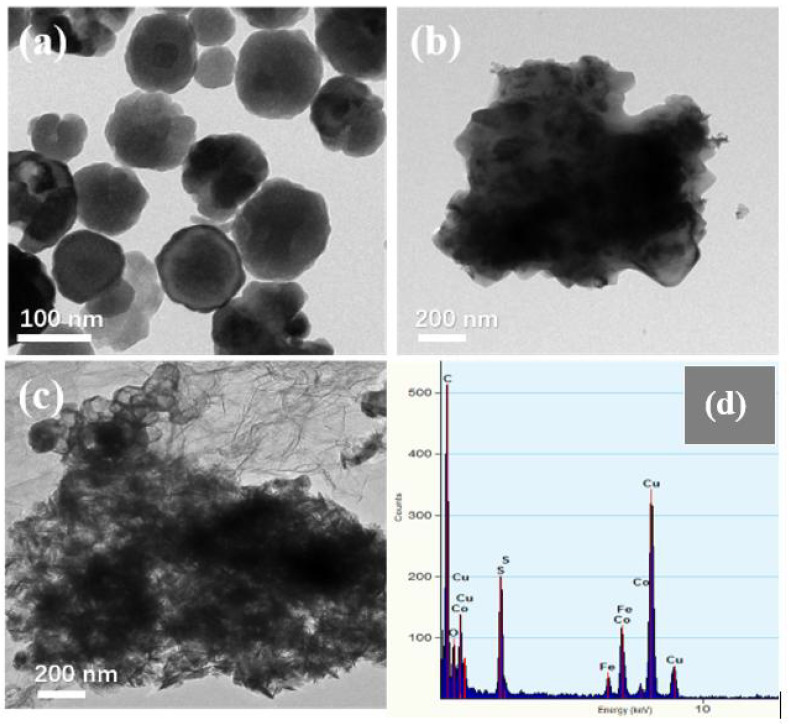
Transmission electron microscopy (TEM) images of Fe_3_O_4_ (**a**), CoS (**b**), CoS@Fe_3_O_4_@rGO (**c**), and its corresponding energy-dispersive X-ray spectrometry image (**d**).

**Figure 5:**
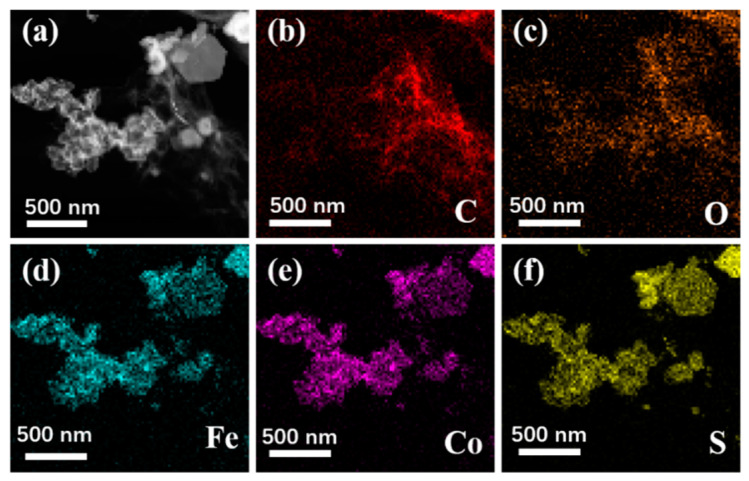
TEM of CoS@Fe_3_O_4_@rGO (**a**) and its corresponding elemental mapping images of C (**b**), O (**c**), Fe (**d**), Co (**e**), and S (**f**).

Figure 3 shows the SEM images of Fe_3_O_4_, CoS@Fe_3_O_4_ and CoS@Fe_3_O_4_@rGO composites. In Figure 3a, there is obvious agglomeration in the Fe_3_O_4_ nanospheres, whose average diameter is approximately 121–158 nm. Figure 3b shows the morphology of CoS, and the CoS samples present a hierarchical spherical structure with a diameter of approximately 2.8 μm. In Figure 3c, the CoS samples are comprised of some interlaced and stacked nanosheets, embedded with many Fe_3_O_4_ microspheres. As shown in Figure 3d, the rGO displays a loosely porous structure with Fe_3_O_4_ and CoS@Fe_3_O_4_ microspheres distributed on it. The unique geometrical structure can not only reduce the density of the composite, but can also increase the attenuation of EMWs in the material. To further study the microstructures of the materials, the TEM images and corresponding EDS image can be seen in Figure 4. As shown in Figure 4a, most Fe_3_O_4_ nanoparticles display a regular spherical shape and a few have a hollow structure. In Figure 4b, the CoS presents an irregular spherical solid structure. From Figure 4c, the formation of the ultra-thin rGO nanosheets can be confirmed with CoS@Fe_3_O_4_ microspheres firmly adhering to them. The corresponding EDS image is shown in Figure 4d, and no other impurity elements can be detected. Combined with the element mappings in Figure 5, the C, O, Fe, Co, and S elements are uniformly distributed in the sample, indicating the successful formation of a CoS@Fe_3_O_4_@rGO aerogel.

### 3.2. Electromagnetic Parameters and Absorption Capability

**Figure 6:**
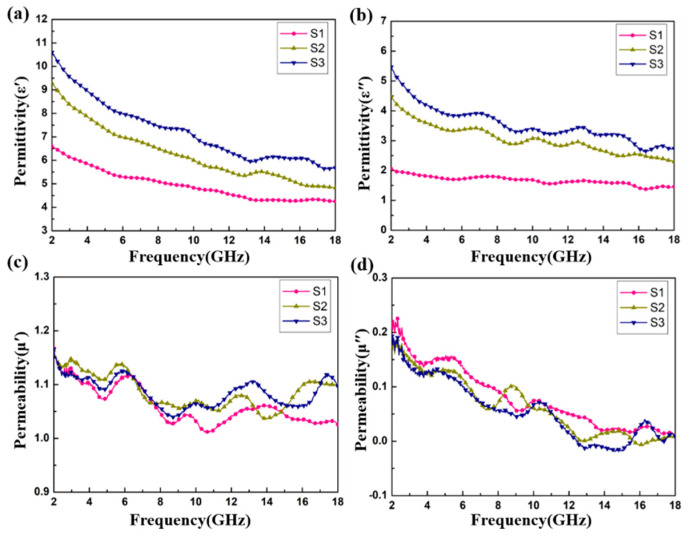
Complex permittivity and permeability of S1, S2, and S3: (**a**) ε′, (**b**) ε″, (**c**) μ′, and (**d**) μ″.

**Figure 7:**
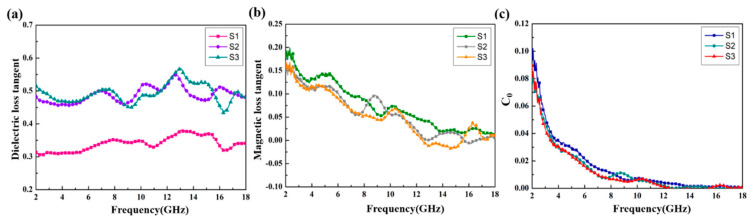
(**a**) Dielectric loss tangent, (**b**) magnetic loss tangent, and (**c**) C_0_–f curves of the samples.

Figure 6 shows the relative complex permittivity (ε_r_ = ε′ − jε″) and the relative complex permeability (μ_r_ = μ′ − jμ″) of the CoS@Fe_3_O_4_@rGO aerogel at different filler loadings with paraffin. The mixtures were made into a coaxial ring and the electromagnetic parameters were measured in the frequency range of 2–18 GHz and calculated by the standard Nicolson–Ross–Weir theory. In Figure 6a,b, the real part of the complex permittivity (ε′) values decrease gradually with the increasing frequency, while the imaginary part of the complex permittivity (ε″) value curves decrease with some fluctuations. Compared to S1 and S2, S3 shows the highest ε′ value (10.5) and ε″ value (5.4), indicating that it exhibits excellent energy storage and dielectric loss properties. As regards Figure 6c,d, the μ′ curves fluctuate between 1.16 and 1.02, while the μ″ curves show a decreasing trend from 0.218 to 0.009. To better evaluate the microwave absorption capability of the CoS@Fe_3_O_4_@rGO aerogel, the dielectric loss tangent ($tan\;\delta_{\varepsilon }=\varepsilon \prime \prime /\varepsilon \prime$) curves and the magnetic loss tangent ($tan\;\delta_{\mu }=\mu \prime \prime /\mu \prime$) curves are given in Figure 7. The $tan\;\delta_{\varepsilon }$ values of S2 and S3 fluctuate between 0.57 and 0.43, and the curves show some obvious resonance peaks at 9–18 GHz, which could be due to the superior dielectric loss property of rGO. It can be speculated that S2 and S3 have better microwave absorption performance. In Figure 7b, the three $tan\;\delta_{\mu }$ curves show a downward trend, overlapping with each other. The magnetic loss mainly includes eddy current loss, natural resonance loss, hysteresis loss, and domain wall resonance loss [49]. Due to the domain wall, resonance losses usually occur in the low frequency range (1–100 MHz) and the effect of hysteresis losses can be negligible in the weak magnetic field; therefore, it can be concluded that eddy current losses and natural resonance losses play a major role in the magnetic loss of materials [31]. The Eddy current loss in a material is usually characterized by the eddy current loss coefficient (C_0_ $=\mu^{\prime \prime }{\left(\mu^{\prime }\right)}^{-2}f^{-1}$), and when eddy current loss occurs inside the materials, C_0_ should be a constant [50]. However, in Figure 7c, the C_0_ curves decrease with the frequency, indicating that the major source of magnetic loss in the CoS@Fe_3_O_4_@rGO aerogel is natural resonance.

**Figure 8:**
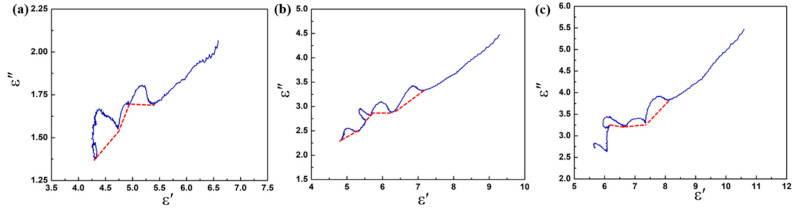
Cole–Cole plots of (**a**) S1, (**b**) S2, and (**c**) S3.

Based on the Debye theory [51], the relaxation polarization process in dielectric materials can be characterized by the plots of ε″ versus ε′ (Cole–Cole semicircle). Generally, every single Cole–Cole semicircle corresponds to a polarization process [52]. In Figure 8, several irregular semicircles can be observed in the plots, and these distortions may be related to the multiple polarizations in the composite. Due to the special heterostructure of the CoS@Fe_3_O_4_@rGO aerogel, in alternating EM fields, electrons accumulate at the heterogeneous interface, inducing interfacial polarization. In addition, dipole polarization can be improved by local defects, abundant functional groups, and unsaturated coordination in the CoS@Fe_3_O_4_@rGO aerogel. It is worth noting that the Cole–Cole curves are close to a straight line at a high frequency, suggesting that the conductivity loss has a leading effect on the dielectric loss [53].

**Figure 9:**
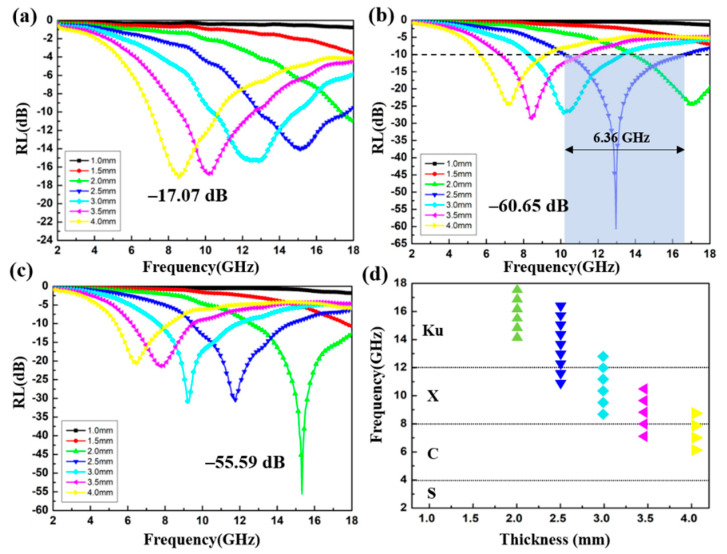
Reflection loss curves of S1 (**a**), S2 (**b**), and S3 (**c**); the effective absorption bandwidth of S2 (**d**).

To investigate the absorption property of the CoS@Fe_3_O_4_@rGO aerogel, the variation of the RL of the products with frequency is shown in Figure 9. Based on the transmission line theory, the value of RL can be calculated by the following equations [54]:

(1) $$Z_{in}=Z_{0}\sqrt{\frac{\mu_{r}}{\varepsilon_{r}}}\tan\;h\left[j\frac{2\pi fd}{c}\sqrt{\mu_{r}\varepsilon_{r}}\right]$$

(2) $$\mathrm{RL}=20\log\left|\frac{Z_{in}-Z_{0}}{Z_{in}+Z_{0}}\right|$$

where *Z*_in_ is the normalized input characteristic impendence, *Z*_0_ is the impendence of air, and *d* represents the thickness of the absorbers. As shown in Figure 9a, when the doping ratio is 4 wt%, the optimal RL of S1 reaches −17.07 dB at 4.00 mm. Although the effective absorption bandwidth (RL < −10 dB) can reach 3.6 GHz (7.04–10.64 GHz), the absorber thickness cannot meet the absorption requirement of light-weight materials. In contrast, S2 exhibits superior microwave absorption capability. The RL value curves of S2 are given in Figure 9b and the corresponding effective absorption bandwidth at different thicknesses is shown in Figure 9d. It can be observed that the optimal RL of S2 is −60.65 dB with an impressive absorption bandwidth of 6.36 GHz (10.24–16.6 GHz) at a 2.5 mm absorber thickness. Moreover, the filler loading of S2 is just 6 wt%, suggesting that the CoS@Fe_3_O_4_@rGO aerogel (6 wt%) can be used as an excellent absorption composite with the advantages of strong absorption, lightweight, and ultra-wide bandwidth. As regards Figure 9c, the minimum RL value of −55.59 dB of S3 can be obtained at 2.0 mm, along with a proficiency absorption bandwidth of 5.4 GHz (12.6–18 GHz). Table 1 shows the comparison of the EMW absorption capability of the CoS@Fe_3_O_4_@rGO aerogel (S2) with previously reported ferrite–graphene-based composites. It is obvious that the CoS@Fe_3_O_4_@rGO aerogel (S2) is superior to similar absorbers in terms of absorption strength, absorption bandwidth, and filler loadings, and it has a promising application potential in the EMW absorption field.

In order to further comprehensively evaluate the EMW absorption capability of the products, the attenuation constant (α) was calculated as follows [59]:

(3) $$\;\alpha =\frac{\sqrt{2}\pi f}{c}\times \sqrt{\left(\mu^{\prime \prime }\varepsilon^{\prime \prime }-\mu^{\prime }\varepsilon^{\prime }\right)+\sqrt{{\left(\mu^{\prime \prime }\varepsilon^{\prime \prime }-\mu^{\prime }\varepsilon^{\prime }\right)}^{2}+{\left(\mu^{\prime }\varepsilon^{\prime \prime }+\mu^{\prime \prime }\varepsilon^{\prime }\right)}^{2}}}$$

**Figure 10:**
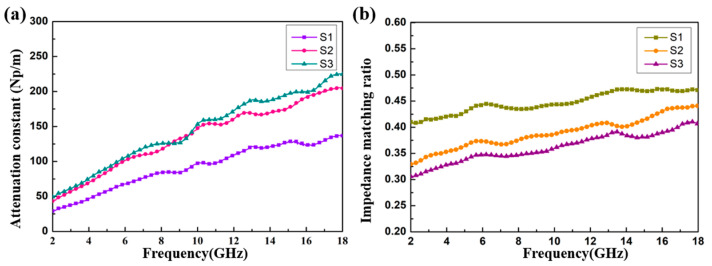
(**a**) The attenuation constant and (**b**) impedance matching ratio of the samples.

It can be seen from Figure 10a that with an increase in the filler loading ratio from 4 to 8 wt%, the attenuation ability of the CoS@Fe_3_O_4_@rGO aerogel can be enhanced significantly. Compared to S1, S2 and S3 exhibit better attenuation ability, which may be attributed to the improved conductivity and magnetic losses. In addition, the impedance matching ratio Z (Z = Z_in_ / Z_0_) curves of the CoS@Fe_3_O_4_@rGO aerogel are shown in Figure 10b. The closer the Z value is to 1, the more EMWs can be incident to the material. In Figure 10b, the impedance matching curve of S3 is lower than S1 and S2, which may be due to the high conductivity and the influence of the skin effect [60]. The oscillating skin current enhances the reflected waves, which is not conductive to the attenuation of EMWs. From Figure 10, it can be confirmed that the excellent attenuation capability and good impedance matching are responsible for the outstanding EMW absorption performance of S2. 

**Figure 11:**
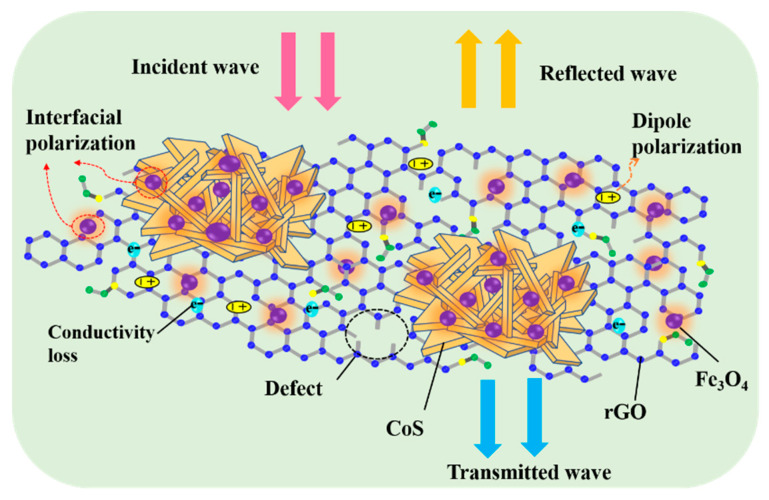
Mechanism illustration of the microwave absorption of the CoS@Fe_3_O_4_@rGO aerogel.

An illustration of the mechanism of microwave absorption for the CoS@Fe_3_O_4_@rGO aerogel is shown in Figure 11. First, the combination of the heterostructure of magnetic Fe_3_O_4_ nanoparticles, CoS, and rGO optimizes the impedance matching of the composite, which provides a prerequisite for EMWs to enter the interior of the composite for further consumption. Next, CoS—modified by Fe_3_O_4_ nanoparticles—introduces a large number of heterointerfaces. More charges accumulate at various heterogeneous interfaces as if they were moving in a capacitor structure, which may be beneficial to enhancing interface polarization [61]. However, the loose 3D porous structure not only reduces the density of the products, but also facilitates the attenuation of EMWs. During multiple reflections and scatterings in the 3D porous structure, the EM energy is converted into heat energy through a longer transmission path [62]. Furthermore, the CoS@Fe_3_O_4_@rGO aerogel can offer an extremely large surface area with abundant functional groups and defects on it, in which many dipoles are generated and the dipole polarization is enhanced [63]. Finally, owing to the excellent dielectric properties of rGO and CoS, the hopping of electrons forms an induced current in an alternating electric field, forming a 3D conductive network, which may be beneficial for improving conduction loss and accelerating the EM attenuation. As a consequence, the CoS@Fe_3_O_4_@rGO aerogel exhibits excellent EMW absorption performance. 

## 4. Conclusions

In this work, a unique CoS@Fe_3_O_4_@rGO aerogel was synthesized using the solvothermal method. The microstructure, elemental composition, EMW absorption performance, and possible mechanism of the products were carefully investigated. The high conductivity of graphene was modulated by the heterogeneous components, and the impedance matching was optimized. The unique porous structure of the CoS@Fe_3_O_4_@rGO aerogel prolonged the propagation path of the electromagnetic waves inside the material, which was conducive to the dissipation of electromagnetic waves through scattering and reflection. As a result, the as-prepared CoS@Fe_3_O_4_@rGO aerogel exhibited a superior microwave-absorbing property, and when the doping ratio was just 6 wt%, the optimal RL of the CoS@Fe_3_O_4_@rGO aerogel could reach −60.65 dB with an ultra-wide bandwidth of 6.36 GHz (10.24–16.6 GHz) at a 2.5 mm thickness. Due to the advantages of excellent dielectric loss and magnetic loss, lightweight, strong absorption, and ultra-wide bandwidth, the CoS@Fe_3_O_4_@rGO aerogel possesses great potential in the field of EMW absorption.

## Figures and Tables

**Table 1:** A comparison of the electromagnetic wave (EMW) absorption capability with some previously reported ferrite–graphene-based composites.

Absorber	Filler Loadings (wt%)	RL_min_ (dB)	Effective Bandwidth (GHZ)	Thickness (mm)	Refs
Fe_3_O_4_/rGO	4	−45	3.2	3.5	[30]
CoS/RGO	20	−54.2	4.0	4.0	[37]
Fe_3_O_4_@rGO	50	−49.8	3.3	3.0	[55]
ZnFe_2_O_4_@RGO@CuS	20	−55.4	7.5	2.2	[56]
Fe_3_O_4_@LAS/rGO	50	−65.0	4.0	2.1	[57]
CoFe_2_O_4_/N–rGO	20	−60.4	6.48	2.2	[58]
CoS@Fe_3_O_4_@rGO	6	−60.65	6.36	2.5	This work

## References

[1] Che R.C., Peng L.-M., Duan X., Chen Q., Liang X.L. (2004). Microwave Absorption Enhancement and Complex Permittivity and Permeability of Fe Encapsulated within Carbon Nanotubes. Adv. Mater..

[2] Landy N.I., Sajuyigbe S., Mock J.J., Smith D.R., Padilla W.J. (2008). Perfect Metamaterial Absorber. Phys. Rev. Lett..

[3] Watts C.M., Liu X., Padilla W.J. (2012). Metamaterial Electromagnetic Wave Absorbers. Adv. Mater..

[4] Cao M.-S., Song W.-L., Hou Z.-L., Wen B., Yuan J. (2010). The effects of temperature and frequency on the dielectric properties, electromagnetic interference shielding and microwave-absorption of short carbon fiber/silica composites. Carbon.

[5] Zhang Y., Huang Y., Zhang T., Chang H., Xiao P., Chen H., Huang Z., Chen Y. (2015). Broadband and Tunable High-Performance Microwave Absorption of an Ultralight and Highly Compressible Graphene Foam. Adv. Mater..

[6] Wen B., Cao M.-S., Lu M., Cao W., Shi H., Liu J., Wang X., Jin H., Fang X., Wang W. (2014). Reduced Graphene Oxides: Light-Weight and High-Efficiency Electromagnetic Interference Shielding at Elevated Temperatures. Adv. Mater..

[7] Zhang X., Dong X.L., Huang H., Liu Y.Y., Wang W.N., Zhu X.G., Lv B., Lei J., Lee C.G. (2006). Microwave absorption properties of the carbon-coated nickel nanocapsules. Appl. Phys. Lett..

[8] Sun G., Dong B., Cao M., Wei B., Hu C. (2011). Hierarchical Dendrite-Like Magnetic Materials of Fe_3_O_4_, γ-Fe_2_O_3_, and Fe with High Performance of Microwave Absorption. Chem. Mater..

[9] Qin F., Brosseau C. (2012). A review and analysis of microwave absorption in polymer composites filled with carbonaceous particles. J. Appl. Phys..

[10] Cao M.-S., Yang J., Song W.-L., Zhang D.-Q., Wen B., Jin H.-B., Hou Z.-L., Yuan J. (2012). Ferroferric Oxide/Multiwalled Carbon Nanotube vs Polyaniline/Ferroferric Oxide/Multiwalled Carbon Nanotube Multiheterostructures for Highly Effective Microwave Absorption. ACS Appl. Mater. Interfaces.

[11] Liu Q., Cao Q., Bi H., Liang C., Yuan K., She W., Yang Y., Che R. (2015). CoNi@SiO_2_@TiO_2_ and CoNi@Air@TiO_2_ Microspheres with Strong Wideband Microwave Absorption. Adv. Mater..

[12] Du Y., Liu W., Qiang R., Wang Y., Han X., Ma J., Xu P. (2014). Shell Thickness-Dependent Microwave Absorption of Core–Shell Fe_3_O_4_@C Composites. ACS Appl. Mater. Interfaces.

[13] Liu J., Che R., Chen H., Zhang F., Xia F., Wu Q., Wang M. (2012). Microwave Absorption Enhancement of Multifunctional Composite Microspheres with Spinel Fe_3_O_4_ Cores and Anatase TiO_2_ Shells. Small.

[14] Singh K., Ohlan A., Pham V.H., Balasubramaniyan R., Varshney S., Jang J., Hur S.H., Choi W.M., Kumar M., Dhawan S.K. (2013). Nanostructured graphene/Fe_3_O_4_ incorporated polyaniline as a high performance shield against electromagnetic pollution. Nanoscale.

[15] Zhu C.L., Zhang M.L., Qiao Y.J., Xiao G., Zhang F., Chen Y.J. (2010). Fe_3_O_4_/TiO_2_ Core/Shell Nanotubes: Synthesis and Magnetic and Electromagnetic Wave Absorption Characteristics. J. Phys. Chem. C.

[16] Chen Y.J., Xiao G., Wang T.S., Ouyang Q.Y., Qi L.H., Ma Y., Gao P., Zhu C.L., Cao M.S., Jin H.B. (2011). Porous Fe_3_O_4_/Carbon Core/Shell Nanorods: Synthesis and Electromagnetic Properties. J. Phys. Chem. C.

[17] Sun X.D., Ma G.Y., Lv X.L., Sui M.X., Li H.B., Wu F., Wang J.J. (2018). Controllable Fabrication of Fe_3_O_4_/ZnO Core-Shell Nanocomposites and Their Electromagnetic Wave Absorption Performance in the 2–18 GHz Frequency Range. Materials.

[18] Wang L., Huang Y., Sun X., Huang H., Liu P., Zong M., Wang Y. (2014). Synthesis and microwave absorption enhancement of graphene@Fe_3_O_4_@SiO_2_@NiO nanosheet hierarchical structures. Nanoscale.

[19] Rubrice K., Castel X., Himdi M., Parneix P. (2016). Dielectric Characteristics and Microwave Absorption of Graphene Composite Materials. Materials.

[20] Li Y., Li D., Yang J., Luo H., Chen F., Wang X., Gong R.Z. (2018). Enhanced Microwave Absorption and Surface Wave Attenuation Properties of Co_0.5_Ni_0.5_Fe_2_O_4_ Fibers/Reduced Graphene Oxide Composites. Materials.

[21] Xu Y., Li J., Huang W. (2017). Porous Graphene Oxide Prepared on Nickel Foam by Electrophoretic Deposition and Thermal Reduction as High-Performance Supercapacitor Electrodes. Materials..

[22] Wong C.P.P., Lai C.W., Lee K.M., Hamid S.B.A. (2015). Advanced Chemical Reduction of Reduced Graphene Oxide and Its Photocatalytic Activity in Degrading Reactive Black 5. Materials..

[23] Zhang N., Huang Y., Zong M., Ding X., Li S., Wang M. (2017). Synthesis of ZnS quantum dots and CoFe_2_O_4_ nanoparticles co-loaded with graphene nanosheets as an efficient broad band EM wave absorber. Chem. Eng. J..

[24] Chandra V., Park J., Chun Y., Lee J.W., Hwang I.-C., Kim K.S. (2010). Water-Dispersible Magnetite-Reduced Graphene Oxide Composites for Arsenic Removal. ACS Nano.

[25] Liu S., Zeng T.H., Hofmann M., Burcombe E., Wei J., Jiang R., Kong J., Chen Y. (2011). Antibacterial Activity of Graphite, Graphite Oxide, Graphene Oxide, and Reduced Graphene Oxide: Membrane and Oxidative Stress. ACS Nano.

[26] Robinson J.T., Tabakman S.M., Liang Y., Wang H., Casalongue H.S., Vinh D., Dai H. (2011). Ultrasmall Reduced Graphene Oxide with High Near-Infrared Absorbance for Photothermal Therapy. J. Am. Chem. Soc..

[27] Zhang N., Huang Y., Wang M. (2018). 3D ferromagnetic graphene nanocomposites with ZnO nanorods and Fe_3_O_4_ nanoparticles co-decorated for efficient electromagnetic wave absorption. Compos. Part B Eng..

[28] Zhang Y., Liu S., Li P., Liao Q., Zhao Y. (2015). Investigation on the optimization, design and microwave absorption properties of reduced graphene oxide/tetrapod-like ZnO composites. Rsc Adv..

[29] Li Z., Li X., Zong Y., Tan G., Sun Y., Lan Y., He M., Ren Z., Zheng X. (2017). Solvothermal synthesis of nitrogen-doped graphene decorated by superparamagnetic Fe_3_O_4_ nanoparticles and their applications as enhanced synergistic microwave absorbers. Carbon.

[30] Zhu L.Y., Zeng X.J., Li X.P., Yang B., Yu R.H. (2017). Hydrothermal synthesis of magnetic Fe_3_O_4_/graphene composites with good electromagnetic microwave absorbing performances. J. Magn. Magn. Mater..

[31] Zhu Y., Murali S., Cai W., Li X., Suk J.W., Potts J.R., Ruoff R.S. (2010). Graphene and Graphene Oxide: Synthesis, Properties, and Applications. Adv. Mater..

[32] Marcano D.C., Kosynkin D.V., Berlin J.M., Sinitskii A., Sun Z., Slesarev A., Alemany L.B., Lu W., Tour J.M. (2010). Improved Synthesis of Graphene Oxide. ACS Nano.

[33] Quan L., Qin F., Estevez D., Wang H., Peng H. (2017). Magnetic graphene for microwave absorbing application: Towards the lightest graphene-based absorber. Carbon.

[34] Li B.Z., Weng X.D., Sun X.D., Zhang Y., Lv X.L., Gu G.X. (2018). Facile synthesis of Fe_3_O_4_/reduced graphene oxide/polyvinyl pyrrolidone ternary composites and their enhanced microwave absorbing properties. J. Saudi Chem. Soc..

[35] Andjelkovic I., Tran D.N.H., Kabiri S., Azari S., Markovic M., Losic D. (2015). Graphene Aerogel Decorated with α-FeOOH Nanoparticles for Efficient Adsorption of Arsenic from Contaminated Waters. ACS Appl. Mater. Interfaces.

[36] Vinh N.T., Tuan L.A., Vinh L.K., Quy N.V. (2020). Synthesis, characterization, and gas sensing properties of Fe_3_O_4_/FeOOH nanocomposites for a mass-type gas sensor. Mater. Sci. Semicond. Process..

[37] Huang T., He M., Zhou Y., Li S., Ding B., Pan W., Huang S., Tong Y. (2017). Solvothermal fabrication of CoS nanoparticles anchored on reduced graphene oxide for high-performance microwave absorption. Synth. Met..

[38] Casiraghi C., Pisana S., Novoselov K.S., Geim A.K., Ferrari A.C. (2007). Raman fingerprint of charged impurities in graphene. Appl. Phys. Lett..

[39] Ferrari A.C. (2007). Raman spectroscopy of graphene and graphite: Disorder, electron–phonon coupling, doping and nonadiabatic effects. Solid State Commun..

[40] Wu F., Xie A., Sun M., Wang Y., Wang M. (2015). Reduced graphene oxide (RGO) modified spongelike polypyrrole (PPy) aerogel for excellent electromagnetic absorption. J. Mater. Chem. A.

[41] Miao X., Pan K., Wang G., Liao Y., Wang L., Zhou W., Jiang B., Pan Q., Tian G. (2013). Well-Dispersed CoS Nanoparticles on a Functionalized Graphene Nanosheet Surface: A Counter Electrode of Dye-Sensitized Solar Cells. Chem. Eur. J..

[42] Liu K., Wei A., Zhang W., Xiao Z., Zhao Y., Liu J. (2018). Synthesis of vertically aligned CoS prismatic nanorods as counter electrodes for dye-sensitized solar cells. J. Mater. Sci. Mater. Electron..

[43] Su J., Cao M., Ren L., Hu C. (2011). Fe_3_O_4_–Graphene Nanocomposites with Improved Lithium Storage and Magnetism Properties. J. Phys. Chem. C.

[44] Ma M., Li W., Tong Z., Yang Y., Ma Y., Cui Z., Wang R., Lyu P., Huang W. (2020). 1D flower-like Fe_3_O_4_@SiO_2_@MnO_2_ nanochains inducing RGO self-assembly into aerogel for high-efficient microwave absorption. Mater. Des..

[45] Wu Y., Shu R., Zhang J., Sun R., Chen Y., Yuan J. (2019). Oxygen vacancy defects enhanced electromagnetic wave absorption properties of 3D net-like multi-walled carbon nanotubes/cerium oxide nanocomposites. J. Alloy Compd..

[46] Zhao H., Cheng Y., Liang X., Du Y., Ji G. (2018). Constructing Large Interconnect Conductive Networks: An Effective Approach for Excellent Electromagnetic Wave Absorption at Gigahertz. Ind. Eng. Chem. Res..

[47] Zhou W., Cui G., Li L., Zhang Z., Lv X., Wang X. (2020). Polypyrrole Chains Decorated on CoS Spheres: A Core-Shell Like Heterostructure for High-Performance Microwave Absorption. Nanomaterials..

[48] Krishnamoorthy K., Veerasubramani G.K., Kim S.J. (2015). Hydrothermal synthesis, characterization and electrochemical properties of cobalt sulfide nanoparticles. Mater. Sci. Semicond. Process..

[49] Zhang Y., Wang X., Cao M.-S. (2018). Confinedly implanted NiFe_2_O_4_-rGO: Cluster tailoring and highly tunable electromagnetic properties for selective-frequency microwave absorption. Nano Res..

[50] Zheng Y., Wang X., Wei S., Zhang B., Yu M., Zhao W., Liu J. (2017). Fabrication of porous graphene-Fe_3_O_4_ hybrid composites with outstanding microwave absorption performance. Compos. Part A Appl. Sci. Manuf..

[51] Wu T., Liu Y., Zeng X., Cui T.T., Zhao Y.T., Li Y.N., Tong G.X. (2016). Facile Hydrothermal Synthesis of Fe_3_O_4_/C Core-Shell Nanorings for Efficient Low-Frequency Microwave Absorption. ACS Appl. Mater. Interfaces.

[52] Yu L., Yu L., Dong Y., Zhu Y., Fu Y., Ni Q. (2019). Compressible polypyrrole aerogel as a lightweight and wideband electromagnetic microwave absorber. J. Mater. Sci. Mater. Electron..

[53] Zhang M., Zhang J., Lv X., Zhang L., Wei Y., Liu S., Shi Y., Gong C. (2018). How to exhibit the efficient electromagnetic wave absorbing performance of RGO aerogel: Less might be better. J. Mater. Sci. Mater. Electron..

[54] Wang Y., Wu X., Zhang W., Luo C., Li J., Wang Y. (2018). Fabrication of flower-like Ni_0.5_Co_0.5_(OH)_2_@PANI and its enhanced microwave absorption performances. Mater. Res. Bull..

[55] Shu X., Zhou J., Liu Y., Wang Y., Hu B., Jiang Y., Kong L., Zhang T., Song H. (2019). Hollow Fe_3_O_4_ microspheres/graphene composites with adjustable electromagnetic absorption properties. Diam. Relat. Mater..

[56] Wang Y., Gao X., Wu X., Zhang W., Wang Q., Luo C. (2018). Hierarchical ZnFe_2_O_4_@RGO@CuS composite: Strong absorption and wide-frequency absorption properties. Ceram. Int..

[57] Yang Y., Xia L., Zhang T., Shi B., Huang L., Zhong B., Zhang X., Wang H., Zhang J., Wen G. (2018). Fe_3_O_4_@LAS/RGO composites with a multiple transmission-absorption mechanism and enhanced electromagnetic wave absorption performance. Chem. Eng. J..

[58] Wang X.Y., Lu Y.K., Zhu T., Chang S.C., Wang W. (2020). CoFe_2_O_4_/N-doped reduced graphene oxide aerogel for high-performance microwave absorption. Chem. Eng. J..

[59] Lu B., Huang H., Dong X.L., Zhang X.F., Lei J.P., Sun J.P., Dong C. (2008). Influence of alloy components on electromagnetic characteristics of core/shell-type Fe–Ni nanoparticles. J. Appl. Phys..

[60] Zhu W., Zhang L., Zhang W., Zhang F., Li Z., Zhu Q., Qi S.-H. (2019). Facile Synthesis of GNPs@NixSy@MoS_2_ Composites with Hierarchical Structures for Microwave Absorption. Nanomaterials.

[61] Hong C.S., Chu S.Y., Tsai C.C., Su W.C. (2011). Manganese effect on the relaxation behaviors of the space charge polarization in Pb(Fe_2/3_W_1/3_)(0.9)Ti_0.1_O_3_ ceramics. Ceram. Int..

[62] Zhang Y., Huang Y., Chen H., Huang Z., Yang Y., Xiao P., Zhou Y., Chen Y. (2016). Composition and structure control of ultralight graphene foam for high-performance microwave absorption. Carbon.

[63] He J.-Z., Wang X.-X., Zhang Y.-L., Cao M.-S. (2016). Small magnetic nanoparticles decorating reduced graphene oxides to tune the electromagnetic attenuation capacity. J. Mater. Chem. C.

